# Role of Magnetite Nanoparticles Size and Concentration on Hyperthermia under Various Field Frequencies and Strengths

**DOI:** 10.3390/molecules26040796

**Published:** 2021-02-04

**Authors:** Venkatesha Narayanaswamy, Sangaraju Sambasivam, Alam Saj, Sulaiman Alaabed, Bashar Issa, Imaddin A. Al-Omari, Ihab M. Obaidat

**Affiliations:** 1 Department of Geology, United Arab Emirates University, Al-Ain 15551, United Arab Emirates; venkateshnrn@gmail.com; 2 Department of Physics, United Arab Emirates University, Al-Ain 15551, United Arab Emirates; sambaphy@gmail.com (S.S.); saju40005@gmail.com (A.S.); 3 Department of Medical Diagnostic Imaging, College of Health Sciences, University of Sharjah, Sharjah 27272, United Arab Emirates; bissa@sharjah.ac.ae; 4 Department of Physics, Sultan Qaboos University, P.O. Box 36, Muscat PC 123, Oman; ialomari@squ.edu.om

**Keywords:** SAR, magnetite nanoparticles, coprecipitation, hyperthermia, particle concentration

## Abstract

Magnetite (Fe_3_O_4_) nanoparticles were synthesized using the chemical coprecipitation method. Several nanoparticle samples were synthesized by varying the concentration of iron salt precursors in the solution for the synthesis. Two batches of nanoparticles with average sizes of 10.2 nm and 12.2 nm with nearly similar particle-size distributions were investigated. The average particle sizes were determined from the XRD patterns and TEM images. For each batch, several samples with different particle concentrations were prepared. Morphological analysis of the samples was performed using TEM. The phase and structure of the particles of each batch were studied using XRD, selected area electron diffraction (SAED), Raman and XPS spectroscopy. Magnetic hysteresis loops were obtained using a Lakeshore vibrating sample magnetometer (VSM) at room temperature. In the two batches, the particles were found to be of the same pure crystalline phase of magnetite. The effects of particle size, size distribution, and concentration on the magnetic properties and magneto thermic efficiency were investigated. Heating profiles, under an alternating magnetic field, were obtained for the two batches of nanoparticles with frequencies 765.85, 634.45, 491.10, 390.25, 349.20, 306.65, and 166.00 kHz and field amplitudes of 100, 200, 250, 300 and 350 G. The specific absorption rate (SAR) values for the particles of size 12.2 nm are higher than those for the particles of size 10.2 nm at all concentrations and field parameters. SAR decreases with the increase of particle concentration. SAR obtained for all the particle concentrations of the two batches increases almost linearly with the field frequency (at fixed field strength) and nonlinearly with the field amplitude (at fixed field frequency). SAR value obtained for magnetite nanoparticles with the highest magnetization is 145.84 W/g at 765.85 kHz and 350 G, whereas the SAR value of the particles with the least magnetization is 81.67 W/g at the same field and frequency.

## 1. Introduction

Nanoscale material applications in biomedical and health science have advanced rapidly in recent times [1]. Extensive attention is given to magnetic nanoparticles for the potential use as an effective diagnostic and treatment agent in fields such as imaging, drug delivery, magnetic separation, and hyperthermia treatments [2,3]. Water dispersion of superparamagnetic nanoparticles, when subjected to an alternating magnetic field (AMF), generates heat via Néel and Brownian relaxation processes [4]. Néel relaxation occurs by rotation of magnetic moment, whereas Brownian relaxation occurs by means of actual rotation of the particles. These relaxation processes depend on several factors like a magnetic moment, the viscosity of the particle dispersion, and the effective magnetic anisotropy of the magnetic nanoparticles [5,6]. In hyperthermia treatment, the temperature of the tumor is raised above 44 °C for a significant period of time; the increased temperature kills tumor cells and does not affect normal cells [7]. The magnetic nanoparticles used in biological systems should be biocompatible and easily excreted from the body. To achieve this, the particles are usually functionalized with suitable functional groups [8]. The magneto thermic efficiency of the magnetic nanoparticles depends on several factors like shape, size, saturation magnetization, particle–particle interaction, and magnetic anisotropy [9,10,11,12,13]. These properties are tuned by employing suitable synthesis methods. The commonly used and well-recorded methods of magnetic nanoparticle synthesis are coprecipitation, organometallic decomposition, sol–gel, and hydrothermal [14,15,16,17]. The coprecipitation method, in general, produces broad size distribution. The size distribution of the magnetic nanoparticles also has a major role in the heating efficiency of the nanoparticles [18]. The current study addresses the effect of particle size distribution and concentration on the magnetic properties and heat efficiency. The magnetic properties of the ferromagnetic materials are dramatically altered due to the quantum size effect and increased surface to volume ratio when they exist as nanosized particles [19]. With respect to the fundamental scientific investigation as well as technological applications of nanoparticles in general and magnetic nanoparticles, in particular, one aspect that is extremely relevant is the “size-effect”. It has been shown by various reports that within the nanoscale regime, small changes in the size of the nanoparticles produce significant changes in the properties exhibited by the nanoparticles under similar stimulus [20,21]. Furthermore, size adds another variable to be accounted for in any scientific and technological study of a system containing several of these magnetic nanoparticles to predict the functional reliability of the assemblies constituting these nanoparticles.

The heating efficiency of the nanoparticles is usually expressed in terms of the specific absorption rate (SAR), which is defined as the heat amount produced by the unit weight of the magnetic nanoparticles during exposure per unit mass at a given strength and frequency of the AMF.

In the linear response theory (LRT), the specific absorption rate (SAR), which is given in watts per gram, is determined using Equation (1) [22]:

(1) $$SAR\left(f,H\right)=\frac{\pi \mu_{0}\chi^{\prime \prime }H^{2}f}{\rho }$$

Here ρ is the mass density of the magnetic material, $\mu_{0}$ is the permeability of free space, $\chi^{\prime \prime }$ is the imaginary part of the susceptibility χ ($\chi =\chi^{\prime }-i\chi^{\prime \prime }$), and H and f are, respectively, the strength and frequency of the AMF. In the LRT, it is assumed that χ stays constant as H increases (M=χH). It is known that this assumption is valid for very small H values. Thus, in the LRT, the heat dissipation of the MNPs has a linear dependence on the AMF frequency and a quadratic dependence on AMF strength. The imaginary part of the susceptibility, $\chi^{\prime \prime }$ is given by Equation (2). The DC susceptibility ($\chi_{0}$) depends on the saturation magnetization and temperature as defined by Equation (3). The effective magnetic relaxation time (τ) which is the resultant of both Néel and Brownian relaxation time is given by Equation (4) [23]:

(2) $$\chi^{\prime \prime }=\frac{2\pi f\tau }{1+{\left(2\pi f\right)}^{2}}\chi_{0}\;$$

(3) $$\mathrm{Where},\;\chi_{0}=\frac{\mu_{0}M_{s}^{2}V}{k_{B}T}$$

(4) $$\frac{1}{\tau }=\frac{1}{\tau_{N}}+\;\frac{1}{\tau_{B}}$$

where τ is the effective magnetic relaxation time, V is the volume of the NP, and $M_{s}$ is the saturation magnetization.

The Brownian relaxation mechanism,$\;\tau_{B}$ is due to the rotation of the particle itself while the particle’s moment flipping occurs and is given by Equation (5) [7,22]:

(5) $$\tau_{B}=\frac{3\;V_{H}\;\eta }{k_{B\;}T}$$

where $V_{H}$ is the hydrodynamic volume of the particle, and η is the viscosity of the liquid where the particle is immersed.

The Néel relaxation time, $\tau_{N}$ is due to the rotation of the magnetic moment of the MNP and is given by Equation (6) [7,24]:

(6) $$\tau_{N}=\frac{\tau_{0}}{2}\sqrt{\frac{\pi k_{B}T}{KV}}exp\left(\frac{KV}{k_{B}T}\right)$$

where K is the magnetic anisotropy of the MNPs and $\tau_{0}$ is a constant ($\approx 10^{-13}-10^{-9}s$) [25].

Hence, in addition to the field strength and frequency, SAR depends also on other factors such as the size, the saturation magnetization, the magnetic anisotropy of the MNPs, and the viscosity of the liquid containing the MNPs. In addition, changing the concentration of the MNPs will lead to changes in the inter-particle interactions and the effective magnetic anisotropy and thus will lead to changes in SAR values [24]. The core/shell structure of the MNPs also will result in changes in the effective magnetic anisotropy and thus the SAR values [26,27]. The particle size distribution also has an effect on SAR values. For samples of the same average particle size, different size distributions will result in different distributions of particle magnetic moments and magnetic anisotropies, which will result in different inter-particle interactions and different responses to the magnetic field and thus resulting in different SAR values.

To effectively study the role of any of the factors that affect SAR, we need to keep the other factors unchanged. However, it is very evident that this is a very difficult task to achieve experimentally.

In order to accurately study the role of particle size and concentration on hyperthermia, the particle size distribution should be fixed. Although a significant number of studies were reported on the role of particle concentration on SAR, less attention has been paid to the role of size distribution [28,29]. In many of the reports, the particle size distribution histogram (obtained from the TEM images) was used to express how narrow the size distribution is and to determine the average size of the particles. However, the nature of the size distribution was not considered. Hence, we believe that more attention should be paid to this issue. In this study, magnetite nanoparticle dispersions with two different average sizes but with very similar particle size distributions were prepared. We report on the role of size and concentration of the nanoparticles on SAR under various AMF parameters. We present a simple demonstration of the effect of particle concentration on SAR at various field strengths and frequencies. Our results provide an accurate estimation of the role of particle concentration on SAR since the role of size distribution was nearly eliminated.

## 2. Results and Discussion

### 2.1. Structural and Magnetic Characterization

**Figure 1:**
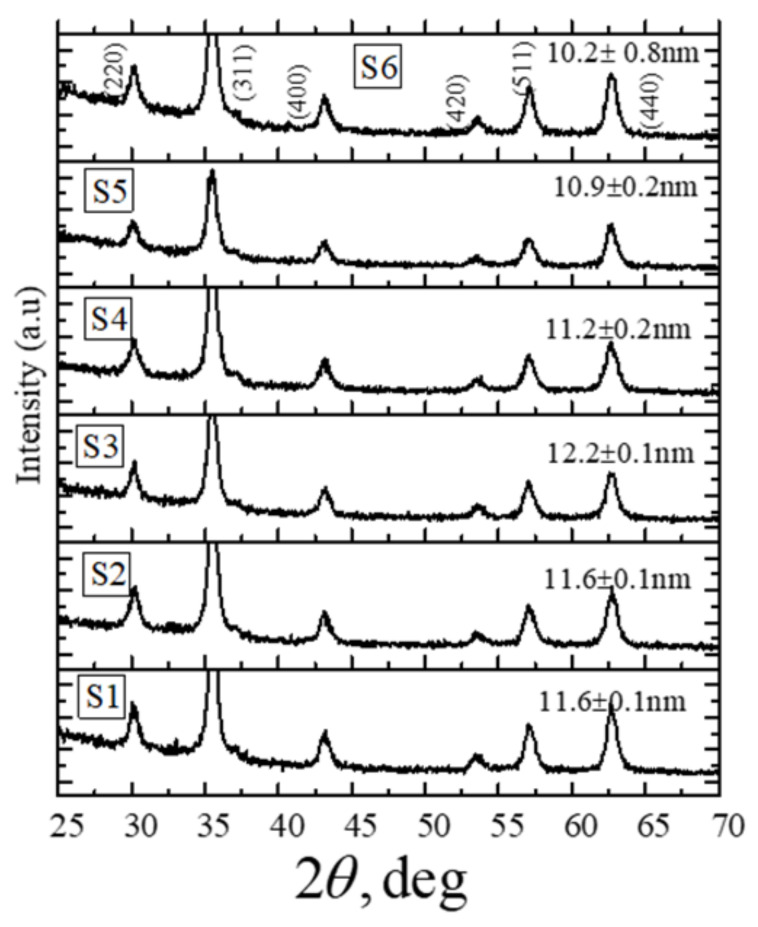
X-ray diffraction patterns of Fe_3_O_4_ nanoparticles synthesized by using the different initial volumes of solution mixtures. The Scherrer average crystallite sizes for nanoparticles are shown above the respective XRD profile.

The XRD profiles for the nanoparticles synthesized from six different solution volumes (75, 100, 150, 200, 250, and 300 mL) of ferric chloride (FeCl_3_) and ferrous chloride tetrahydrate (FeCl_2_.4H_2_O) salt solutions in distilled water are shown in Figure 1. The nanoparticles are named as shown in Table 1. The crystallite sizes are indicated above the XRD profiles, and sizes are also listed in Table 1, along with the initial volume of the solution mixtures used for the synthesis. The XRD profiles in Figure 1 indicate that the as-synthesized nanoparticles contain only pure Fe_3_O_4_ phase [30]. For all six cases, the average crystallite size was obtained from the full width at half maximum of the (311) Fe_3_O_4_ reflection in the XRD profile using the Scherer formula [31]. It can be seen from Table 1 that the average sizes for the six sets of nanoparticles obtained from the XRD profiles have a nonmonotonic behavior with the initial volume of the solution mixtures used for the synthesis. The minimum particle size is 10.2 nm (S6), and the largest particle size is 12.2 nm (S3), which are listed in Table 1.

The difference in the sizes of the particles can be attributed to the iron salt concentration-dependent competing/co-occurring nucleation and growth processes and the electrostatic repulsion effect during the particle formation reaction. According to the Lamer diagram that illustrates the formation of particles involving the nucleation and crystal growth mechanisms, a high supersaturation is required to initiate the nucleation, but growth can occur at a relatively lower degree of supersaturations [32]. Therefore, for solutions where the concentration of iron salt is high, more than one successive nucleation bursts and simultaneous particle growth can happen. In contrast to the case of solutions with lower concentrations of iron salt, a comparatively smaller number of nucleation events will happen as an initial burst of nucleation may lower the concentration below the level required for another successive nucleation event. A high concentration of ions in the initial solution can produce smaller particle sizes due to higher electrostatic repulsion for the species migrating towards a growing particle. Whereas at lower concentrations of ions, the electrostatic repulsion factor is relatively less dominant, and the particle growth in such cases is primarily limited by the diffusion of species from solution to the growing surface of a particle.

**Figure 2:**
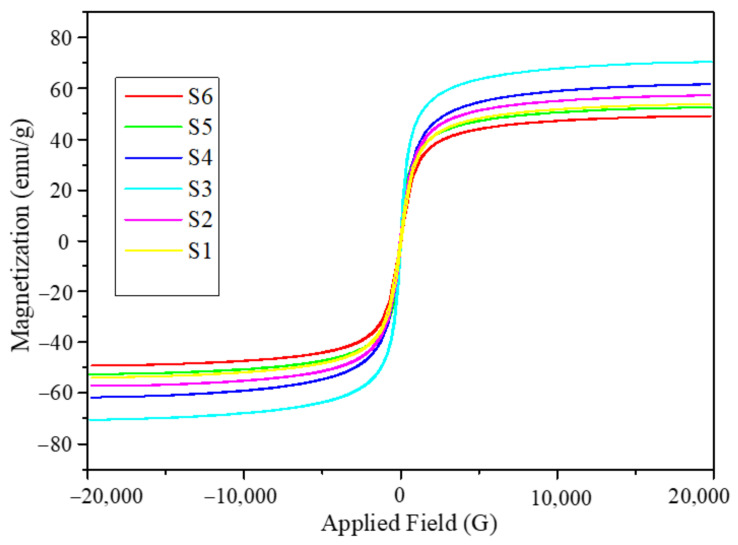
Magnetic hysteresis loops obtained at room temperature in the field range of −2 T to +2 T for Fe_3_O_4_ six sets of nanoparticles.

The magnetic hysteresis loops obtained for the Fe_3_O_4_ nanoparticles synthesized from the six different solutions with different initial volumes of solution mixtures are shown in Figure 2. The magnetic hysteresis loops were obtained at room temperature by applying a magnetic field within the range of -2 T to +2 T. The magnetic response curves in Figure 2 reveal that the Fe_3_O_4_ nanoparticles obtained from six different synthetic conditions are superparamagnetic in nature. It can also be seen that saturation magnetization was nearly achieved in all the samples. The values of the mass normalized saturation magnetization, M_s_ of the nanoparticles are listed in Table 2. It can be seen that M_s_ has a nonmonotonic behavior with the volume of the solution mixture used for the synthesis. The M_s_ values listed in Table 2 are in correlation with the crystallite sizes obtained from XRD. For further investigations of the nanoparticles, we consider only two of these six sets of nanoparticles. The batch of nanoparticles with the lowest M_s_ is S6 (49.16 emu/g), while the batch with the highest M_s_ is S3 (70.37 emu/g), corresponding to the initial volume of the solution mixtures 300 and 150 mL, respectively, as listed in Table 1 and Table 2.

**Figure 3:**
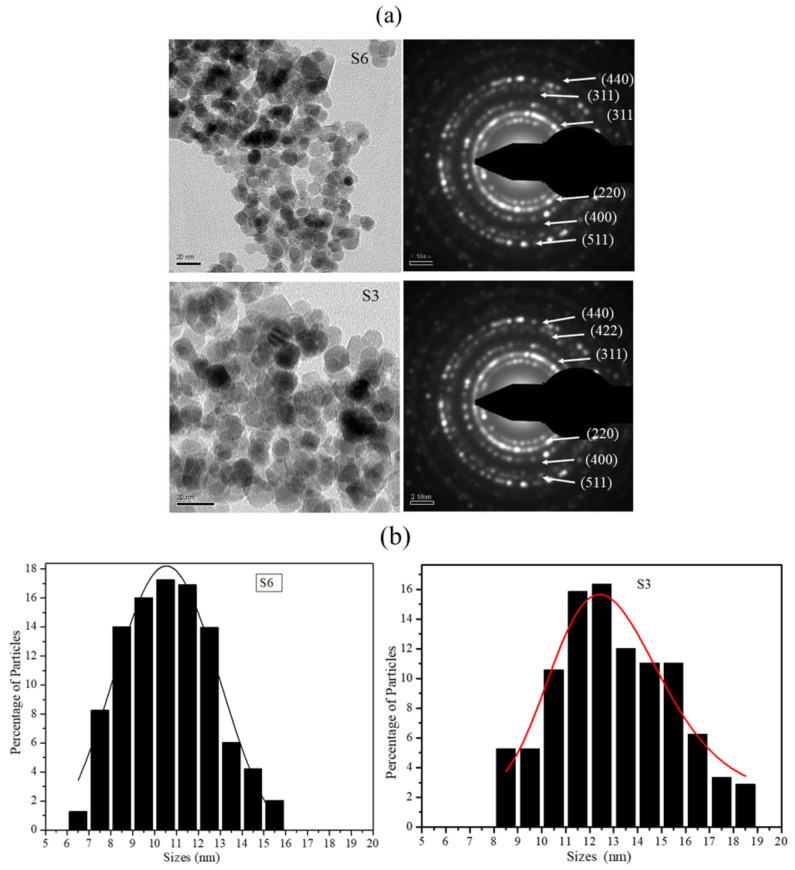
(**a**) TEM images and selected area electron diffraction patterns of batches S6 and S3 of Fe_3_O_4_ nanoparticles. (**b**) Size distribution histogram for S6 and S3 nanoparticle batches.

The as-synthesized S3 (70.37 emu/g) and S6 (49.16 emu/g) nanoparticle samples were imaged in a transmission electron microscope. The representative TEM bright-field micrographs of the samples are shown in Figure 3a. Several bright-field images of 220 nanoparticles (of each batch) were used to obtain the size distribution histograms using sigma scan software. Figure 3b shows percentage size distributions of S3 and S6 nanoparticles. It is observed from Figure 3b that the size distributions of both nanoparticle batches are very similar. The selected area electron diffraction (SAED) pattern obtained from both batches of particles is also provided in Figure 3a. SAED patterns again confirmed the presence of only magnetite phase in the two batches of the nanoparticles [26].

### 2.2. Raman and XPS Analysis of Ferrite Nanoparticles

**Figure 4:**
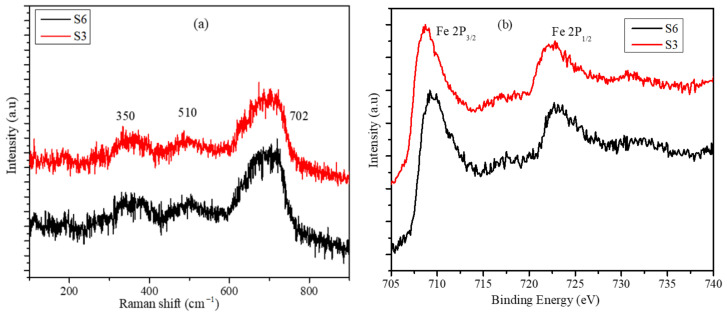
(**a**) Raman spectroscopy, and (**b**) XPS-binding energy profiles of S6 and S3 samples.

The presence of only magnetite phase in S3 and S6 nanoparticle batches were further confirmed from the Raman spectroscopy analysis. In the Raman spectroscopy profile provided in Figure 4a, the high-intensity peak at 702 cm^−1^ and absence of any peak at 225 cm^−1^, respectively, confirms that only the Fe_3_O_4_ phase is present and the Fe_2_O_3_ phase is absent in both batches [33]. XPS analysis was also performed to confirm the phases and the XPS data is provided in Figure 4b. The overlap of the binding energy profiles in the XPS analysis data provided in Figure 4b again confirms the presence of similar phases in the nanoparticles synthesized with various Fe^3+^ concentrations. The peaks at the binding energy values of 710 and 725 eV correspond to Fe in a magnetite lattice [34]. Note that it is important to confirm the absence of the Fe_2_O_3_ phase in the nanoparticles as it can lead to the reduction in the mass magnetization value obtained from the dispersion of nanoparticles.

The analysis of data obtained from the XRD profiles, magnetic measurements, XPS, Raman spectroscopy and the size distribution histograms analysis clearly illustrated that the difference in the magnetic response of the magnetite nanoparticles obtained from the two batches with similar magnetic and structural phases and with very similar size distributions is primarily due to the difference in the average particle sizes. For an individual nanoparticle, the magnetic behavior essentially depends on the relative response from the magnetic order present on its surface and in its core. The relative volume fraction of the two phases changes drastically with the change in the particle size, and hence the magnetic response such as saturation magnetization varies with size under a similar stimulus. Therefore, for a collection of particles of different sizes, the saturation magnetization value would be dependent on the relative volume fractions of the nanoparticles lying in the different size regimens within the “nanosize” limits. A small percentage of particles in the higher size regime can influence the collective response from the dispersion considerably. Finite-size effects and surface-spin effects have significant roles in the phenomenon of the decrease of the saturation magnetization value in the nanosize regime [35,36,37]. What is particularly intriguing is the fact that the size distribution profiles for the two cases shown in Figure 3a are very similar, which illustrates that very small changes in the particle size are able to generate responses that are distinctly different.

### 2.3. Calorimetric Studies

**Figure 5:**
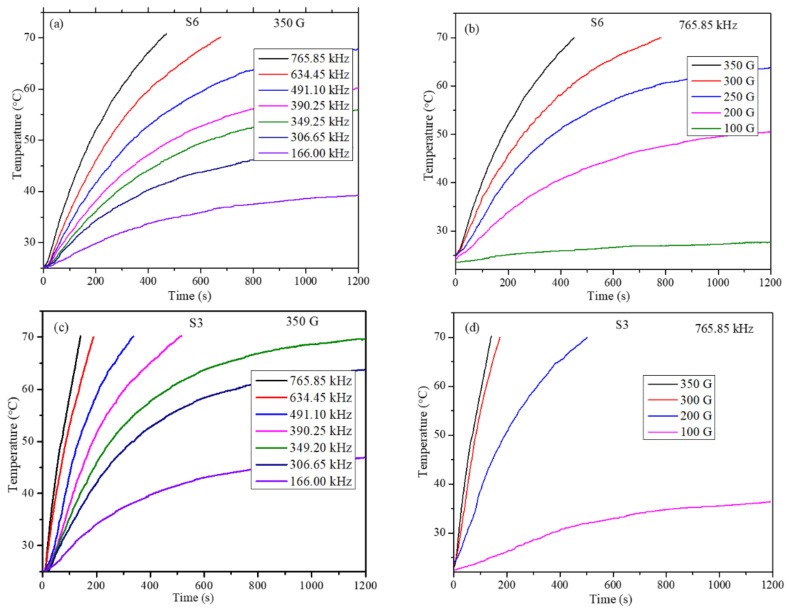
Heating profiles of concentration 10 mg/ml (**a**) for S6 samples at fixed field strength 350 G, (**b**) for S6 samples at fixed AC frequency 765.85 kHz, (**c**) for S3 samples at fixed field strength 350 G, and (**d**) for S3 samples at fixed AC frequency 765.85 kHz

Heating profiles of S6 and S3 nanoparticles were obtained for particle concentration dispersions of 5, 10, 15, 20, and 30 mg/mL. To study the effect of frequency of the AMF on the heating efficiency of nanoparticles, the field strength was kept constant at 350 G while the frequency was varied between 166.00 and 765.86 kHz. Whereas to study the effect of field strength on heating ability, the frequency was fixed at 765.86 kHz while the strength was varied between 100 and 300 G. These instrument parameters are well within the permissible levels (*C* = *H* × *f* = 5 × 10^9^ Am^−1^ s^−1^ (6.25 × 107 Oe Hz), where *H* is the strength and *f* is the frequency of the applied AMF field, respectively, the product should be less than 5 × 10^9^ Am^−1^ s^−1^ to be able to use them in human trials) [38]. The heating profiles for one concentration (10 mg/mL) for samples S6 and S3 are shown in Figure 5. Heating profiles for a given concentration and field parameters were recorded to the dispersion temperature reaches 70 °C. In the cases where the dispersion temperature did not reach 70 °C, the readings were recorded for a maximum of 20 min of exposure time. The heating profiles clearly show that the magneto thermic ability has a strong dependency on particle concentration, strength, and frequency of the AMF field. For most of the particle concentrations at low field frequency, 166.00 kHz and strength of 100 G, the temperature of the particle dispersion could not increase more than 40 °C, which indicates that these parameters are not useful to obtain the required efficiency. The S3 batch of nanoparticles, which has the highest magnetization of 70.37 emu/g, has shown that it crosses 70 °C in a short time and with a small concentration.

**Figure 6:**
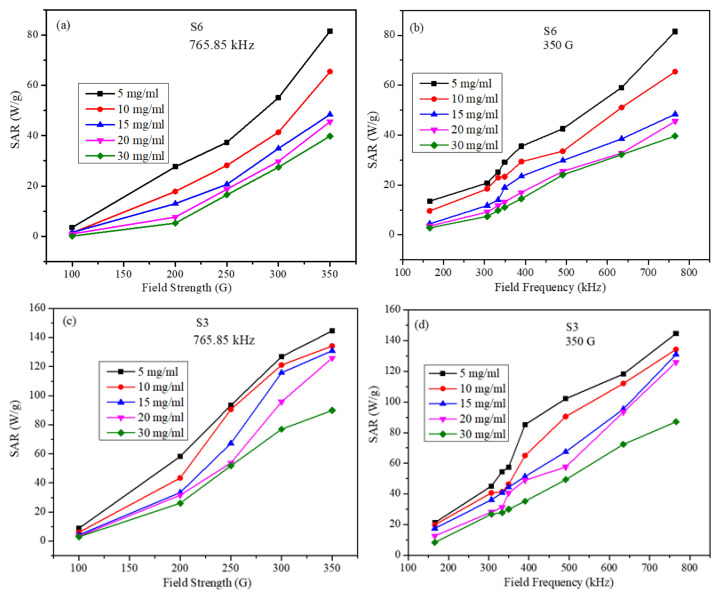
SAR values (**a**) for S6 sample as a function of the field strength at a fixed frequency for several particle concentrations, (**b**) for S6 sample as a function of the field frequency at fixed strength for several particle concentrations, (**c**) for S3 sample as a function of the field strength at a fixed frequency for several particle concentrations, and (**d**) for S3 sample as a function of the field frequency at fixed strength for several particle concentrations (lines in figures are just to guide the eye).

The SAR values for all the samples were calculated using Equation (7). The initial slope of the linear part of the heating curve was used to calculate SAR values. The SAR values calculated for S3 and S6 samples are shown in Figure 6 as a function of the strength and frequency of the AMF for several particle concentrations. For the frequency-dependent measurements, the field amplitude was kept constant at 350 G, whereas for the field strength-dependent measurements, the frequency was maintained at 765.85 kHz. Several observations can be made from the SAR data: (a) sample S3, with the higher saturation magnetization, has a maximum SAR value of 145.85 W/g, whereas the S6 particles have a maximum SAR value of 81.67 W/g; (b) these maximum SAR values were obtained at maximum field frequency and strength for the smallest particle concentration; (c) for both S3 and S6 samples, and for all particle concentrations, SAR values increase almost linearly with the field frequency and nonlinearly with the field strength. In the LRT, the nanoparticles are assumed to be non-interacting single domain particles. In our samples, the particles were PEG-coated, and thus the dipolar inter-particle interactions will be weaker than those for the uncoated particles. Hence, the dependence of SAR on the AMF parameters is not exactly as suggested by the LRT. This slight discrepancy is attributed to the small inter-particle interaction among the particles, which increases with increasing the particle concentration.

**Figure 7:**
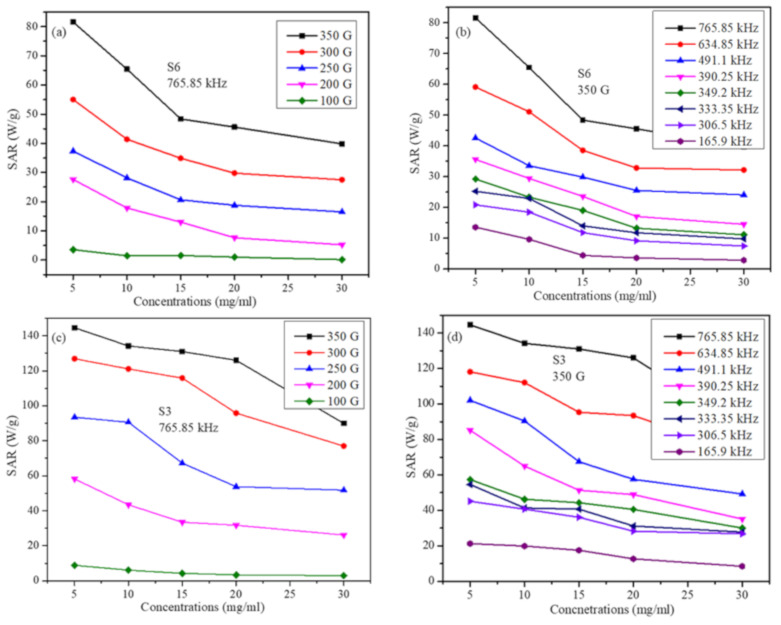
SAR values as a function of the particle concentration (**a**) for S6 sample at a fixed frequency for several field strengths, (**b**) for S6 sample at fixed field strength for several frequencies, (**c**) for S3 sample at a fixed frequency for several field strengths (**d**) for S3 sample at fixed field strength for several frequencies (lines in figures are just to guide the eye).

Figure 7 shows the SAR in both samples as a function of particle concentration at several field parameters. It can be seen in Figure 7 that SAR decreases with particle concentration in both samples and at all field parameters. These results are in accord with some reports [29]. However, the trend of decrease of SAR with particle concentration has roughly two distinct features (these features are more pronounced of sample S6). The first feature is a fast decrease in SAR with increasing particle concentration up to 15 mg/mL. The second feature is the slow and nearly linear decrease of SAR from 15 mg/mL up to 30 mg/mL concentrations. This indicates that for all the particle concentrations and field parameters used in the study, inter-particle interactions have a suppression effect on SAR [39]. However, as the inter-particle interactions become larger, the suppression effect becomes smaller. This means one should be careful when analyzing the role of particle concentration on SAR as it may not be the same at all concentrations. In addition, this behavior may not be the same far as all particle sizes.

**Figure 8:**
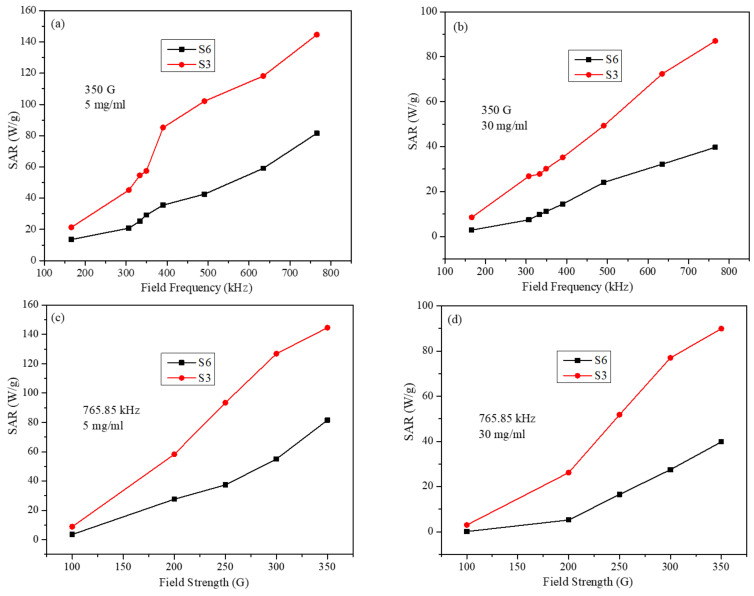
SAR values for S3 and S6 samples (**a**) at fixed field strength, at 5 mg/mL concentration, (**b**) at fixed field strength, at 30 mg/mL concentration, (**c**) at fixed field frequency, at 5 mg/mL, and (**d**) at fixed field frequency, at 30 mg/mL (lines in figures are just to guide the eye).

Figure 8 displays SAR values at the smallest and largest particle concentrations as a function of frequency (at fixed field strength) and as a function of field strength (at a fixed frequency). It can be seen that SAR values for S3 and S6 samples (at the lowest concentration of 5 mg/mL) are very close at the lowest field frequency (Figure 8a) and at the lowest field strength (Figure 8c). However, the difference in SAR values of the two samples becomes bigger as the field strength and frequency are increased. The increase in SAR values with respect to concentrations for S3 is larger than that for S6. The large difference in SAR values occurs at the highest frequency and amplitude values. A similar trend is observed at the highest concentration of 30 mg/mL (Figure 8a,c).

**Figure 9:**
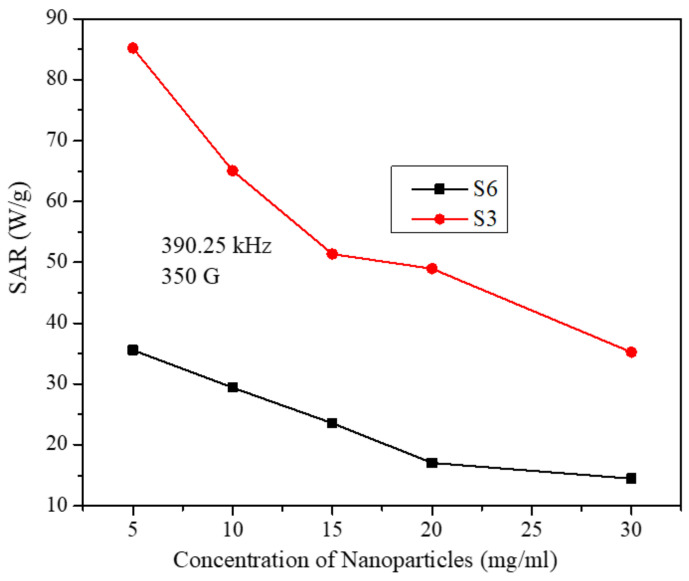
SAR values as a function of particle concentration for samples S3 and S6 at fixed field frequency and strength. (The lines in the figure are just to guide the eye).

Figure 9 displays the SAR values for both samples as a function of particle concentration at a fixed intermediate field frequency of 390.25 kHz and fixed field strength of 350 G. As discussed earlier, SAR values for both samples decrease with increasing the particle concentration. However, SAR values for the S3 sample are larger than those for the S6 sample at all concentrations and thus reflecting the role of particle size on SAR. Similar behavior was observed at all AMF frequencies and strengths.

From Figure 8 and Figure 9, we can see that the SAR values for the S3 sample are always larger than those for the S6 sample at all concentrations and field parameters and thus evidencing the role of size on heating efficiency as suggested by the LRT. It is important to indicate here that when comparing the SAR values with respect to the size of the particles, only the size of the uncoated particles (taken from the TEM images) was considered. This is because the particles of both samples are below 15 nm, below which the effective relaxation time is dominated by the Néel relaxation time [7], and thus, the hydrodynamic volume (which includes the coating material) of the particles was not involved.

## 3. Materials and Methods

### 3.1. Synthesis of the Nanoparticles

Fe_3_O_4_ nanoparticles with different average sizes but with nearly similar particle size distributions were prepared by dissolving ferric chloride (FeCl_3_-6.9960 g) and ferrous chloride tetrahydrate (FeCl_2_.4H_2_O-4.2720 g) salts in deionized water. In order to vary the concentrations of the iron salts in the precursor solutions, the total volume of the solution used for the synthesis was varied starting from 75, 100, 150, 200, 250, and 300 mL while keeping the total amounts and the molar ratio of Fe^2+^/Fe^3+^ equal to 0.5 in all cases. For precipitation of the metal hydroxides, 25% ammonia solution was added at a rate of 4 mL/min into the solution containing the iron salts. The pH of the solution was maintained within the range 11–12 while the solution was subsequently heated in the open air at 65–70 °C for about 30 min to complete the ferrite particle formation. The particles obtained were washed several times with distilled water and dried in an oven before conducting further characterizations. To coat the nanoparticle, 500 mg of Fe_3_O_4_ magnetic nanoparticles synthesized in the previous step were added to 50 mg of PEG solution and sonicated for 60 min at kept at room temperature for 24 h. The excess PEG from the nanoparticle dispersion was removed using centrifugation at 8000 rpm for 15 min. The PEG-coated nanoparticles were redispersed in distilled water by sonicating for 15 min and further used for heating efficiency measurements.

### 3.2. Characterization of the Nanoparticles

Structural phases of the nanoparticles and the crystallite sizes were determined from the X-ray diffraction profile obtained using an X-Pert Pro PANalytical machine employing a Cuk_α_ radiation source. A 300 keV field emission FEI Tecnai F-30 transmission electron microscope (TEM) was used to obtain bright field images, selected area electron diffraction patterns and particle-size distribution information. Samples for the TEM-based analysis were prepared by drop-drying a highly dilute dispersion of the nanoparticles onto an electron transparent carbon-coated Cu grid. Magnetic hysteresis loops were obtained for the nanoparticles using a Lakeshore vibrating sample magnetometer (VSM). Raman spectra from the as-synthesized samples were obtained using a microscope setup (HORIBA JOBIN YVON, Lab RAM H) consisting of a diode-pumped solid-state laser operating at 532 nm with a charge-coupled detector.

### 3.3. Magneto Thermal Measurements

Two batches of two different sizes of the magnetite nanoparticles were prepared and PEG-coated. The nanoparticles were dispersed in water by sonication, after which one mL dispersions of 5, 10, 15, 20, and 30 mg/mL concentration was used for obtaining the heating profiles. The heating profiles of nanoparticles were obtained using a nanoScale Biomagnets hyperthermia instrument. The calorimetric measurements were conducted using an AMF. In one kind of measurement, the field strength was fixed at 350 G for all the field frequencies of 765.85, 634.45, 491.10, 390.25, 349.20, 306.65, and 166.00 kHz. In the second kind of measurements, the field frequency was fixed at 765.85 kHz for all the field strengths of 100, 200, 250, 300 and 350 G. The SAR values for all the concentrations of nanoparticles were evaluated from the slope of the linear part of the heating profile curve according to the Equation (7):

(7) $$SAR(W/g)=\frac{C}{m}\frac{dT}{dt}$$

where C is the specific heat capacity of water (has a value of 4186 JL^−1^ K^−1^ at room temperature), m is the mass concentration of the magnetic material (g/L), and dT/dt is the initial slope of the temperature versus the time curve. This choice was considered because at the initial stage of heating, heat transfer between the sample and the environment will be negligible, and thus, adiabatic conditions are valid. In addition, temperature variations within the sample are expected to very small in the initial heating process and thus can be ignored [40]. After a thorough sonication process of each dispersion, heating profiles are obtained. The slope of the initial heating curve was calculated in the first 20 s, where the particles can be assumed to be will-dispersed without significant precipitation.

## 4. Conclusions

Two batches of magnetite nanoparticles of different average sizes but with very similar particle size distributions were synthesized by the chemical coprecipitation method. XRD patterns and TEM images were used to determine the average size of the particles. Structural and magnetic phases of the two batch were studied using XRD, SAED, Raman and XPS spectroscopy. In the two batches, the particles were found to be of the same pure crystalline phase of magnetite. Calorimetric measurements were performed to measure the specific absorption rate (SAR) of both batches at several particle concentrations and field parameters. We found that SAR shows a high dependency on particle size and concentration, frequency, and field strength. The SAR obtained for all the particle concentrations of the two batches increases almost linearly with the field frequency (at fixed field strength) and nonlinearly with the field strength (at fixed field frequency). The slight discrepancy with LRT is attributed to the existence of inter-particle interactions. Under similar experimental conditions, SAR values of the batch of larger average particle size are always larger than those of the batch of the smaller average particle size, and thus reflecting the role of particle size on SAR. The highest SAR value obtained is 145.84 W/g at 765.85 kHz and 350 G. It was also found that SAR decreases with the increase of particle concentration of both samples, and thus reflecting the suppression role of interparticle interactions on SAR. However, this suppression has different trends as a function of particle concentration in both samples.

## Figures and Tables

**Table 1:** Average particle sizes obtained from XRD for nanoparticle batches synthesized using 75, 100, 150, 200, 250, and 300 mL of the initial volume of solution mixtures.

Initial Volume of the Solution Mixture (mL)	Nanoparticle Batch	Average Scherrer Sizes (nm)
75	S1	11.6 ± 0.1
100	S2	11.6 ± 0.1
150	S3	12.2 ± 0.1
200	S4	11.2 ± 0.2
250	S5	10.9 ± 0.2
300	S6	10.2 ± 0.8

**Table 2:** Mass normalized magnetization (M_s_) values of six sets of Fe_3_O_4_ nanoparticles.

Nanoparticles	S1	S2	S3	S4	S5	S6
Saturation magnetization (emu/g)	53.83	57.17	70.37	61.57	52.45	49.16

## Data Availability

The data presented in this study are available in the article.

## Sample Availability

Samples of the compounds are not available from the authors.
